# Laser Scribing Fabrication of Graphitic Carbon Biosensors for Label-Free Detection of Interleukin-6

**DOI:** 10.3390/nano11082110

**Published:** 2021-08-19

**Authors:** Pei Shee Tan, Eoghan Vaughan, Jahidul Islam, Niall Burke, Daniela Iacopino, Joanna B. Tierney

**Affiliations:** 1 Shannon Applied Biotechnology Centre, Munster Technological University, Tralee, V92KA43 Kerry, Ireland; Pei.Shee.Tan@research.ittralee.ie (P.S.T.); Niall.Burke@staff.ittralee.ie (N.B.); Joanna.Tierney@staff.ittralee.ie (J.B.T.); 2 Department of Biological and Pharmaceutical Sciences, Munster Technological University, Tralee, V92KA43 Kerry, Ireland; 3 Tyndall National Institute, University College Cork, Dyke Parade, T12R5CP Cork, Ireland; eoghan.vaughan@tyndall.ie (E.V.); jahidul.islam@tyndall.ie (J.I.)

**Keywords:** direct laser writing, graphitic carbon, electrochemical immunosensor, IL-6, inflammation, ELISA

## Abstract

Interleukin-6 (IL-6) is an important immuno-modulating cytokine playing a pivotal role in inflammatory processes in disease induction and progression. As IL-6 serves as an important indicator of disease state, it is of paramount importance to develop low cost, fast and sensitive improved methods of detection. Here we present an electrochemical immunosensor platform based on the use of highly porous graphitic carbon electrodes fabricated by direct laser writing of commercial polyimide tapes and chemically modified with capture IL-6 antibodies. The unique porous and 3D morphology, as well as the high density of edge planes of the graphitic carbon electrodes, resulted in a fast heterogeneous electron transfer (HET) rate, k^0^ = 0.13 cm/s. The resulting immunosensor showed a linear response to log of concentration in the working range of 10 to 500 pg/mL, and low limit of detection (LOD) of 5.1 pg/mL IL-6 in phosphate buffer saline. The total test time was approximately 90 min, faster than the time required for ELISA testing. Moreover, the assay did not require additional sample pre-concentration or labelling steps. The immunosensor shelf-life was long, with stable results obtained after 6 weeks of storage at 4 °C, and the selectivity was high, as no response was obtained in the presence of another inflammatory cytokine, Interlukin-4. These results show that laser-fabricated graphitic carbon electrodes can be used as selective and sensitive electrochemical immunosensors and offer a viable option for rapid and low-cost biomarker detection for point-of-care analysis.

## 1. Introduction

Interleukin-6 (IL-6) is a soluble protein secreted from a variety of innate and adaptive immune and non-immune cell types and involved in the modulation of many cellular reactions including sensitization, chemically-induced tissue damage, control of cell replication and apoptosis [1,2]. ILn-6 is best known for its perilous role in generating a variety of inflammatory responses underpinning psoriasis, rheumatoid arthritis, cardiovascular disease, and inflammatory bowel disease [3,4]. IL-6 is relevant to the early diagnosis of many diseases such as diabetes and Alzheimer’s as well as being a biomarker over-expressed by several types of cancer, including head and neck squamous cell carcinoma, breast, oral, cervical and colorectal cancer [5,6,7,8,9]. Recently, IL-6 has been chosen as a potential target for COVID-19 therapy [10]. Due to its potential as an early diagnostic indicator of inflammatory events, sensitive detection of IL-6 is of the utmost importance. However, detection is challenging in situ owing to low IL-6 serum concentrations, lower than 6 pg/mL in healthy humans and reaching elevated values of >80 pg/mL in cases of cancer or abnormal inflammation [11,12].

Traditionally, IL-6 is quantitatively detected in blood by enzyme-linked immunosorbent assays (ELISA). In spite of their extensive use for clinical protein determination and low detection limits (as low as 3 pg/mL for protein biomarkers) such assays suffer limitations in analysis time, sample size, and real-time measurements [13,14]. Other quantitative methods, such as antibody array assays and bead-based assays suffer similar limitations and require costly, high-maintenance instrumentation, specialized infrastructure and highly skilled personnel [15]. As an alternative, a number of electrochemical sensors have been recently developed, showing high sensitivity, strong selectivity and good detection limits [16,17,18,19]. For example, Wang et al. developed a carbon nanotubes (CNTs)-based electrochemical impedance sensor able to detect IL-6 antigen in serum at a low limit of 0.01 fg mL^−1^. The sensor exhibited high stability and selectivity and its sensitivity did not change upon storage at 4 °C, over one month [20]. Tertis et al. developed a label-free electrochemical aptasensor based on gold and polypyrrole nanoparticles able to detect IL-6 over a wide linear range from 1 pg mL^−1^ to 15 mg mL^−1^ with a detection limit of 0.33 pg mL^−1^ [18]. Jensen et al., fabricated gold nanoparticle arrays by inkjet-printing, and demonstrated low cost detection of IL-6 with detection limit of 20 pg mL^−1^ in bovine serum, sensitivity of 11.4 nA pg^−1^ cm^−2^, and a linear dynamic range of 20–400 pg/mL [17]. As well as the direct immunoassays described, a variety of indirect competitive electrochemical, sandwich nanoparticle labelled electrochemical and sandwich enzyme linked electrochemical immunoassays have been developed. A general overview of all recent advances in electrochemical sensing of IL-6 has been recently compiled in a comprehensive review by Khan et al. [21].

Graphene and graphitic materials possess remarkable physical and chemical properties such as a wide electrochemical window and electrocatalytic activity for many redox reactions, which make them particularly suitable for use as electrochemical sensors [22,23,24]. However, standard fabrication techniques of graphene-like electrode materials, including chemical vapour deposition (CVD) [25] and laser ablation [26] are usually costly, time consuming and non-environmentally friendly. Other alternative methods such as screen printing or inkjet printing require the use of binders, additives or post print processes such as laser, thermal or photonic annealing, that affect the electrochemical sensitivity and increase the overall cost of production of the resulting electrodes [27,28,29]. Recently, direct laser writing techniques have been proposed as an alternative for the fabrication of disposable graphene-like electrochemical sensors. In this method, pioneered by the group of Tour et al., a simple computer-aided laser-scribing process enabled fast patterning of a variety of electrode designs on flexible polyimide (Kapton tape) under ambient conditions [30,31]. The capability of laser-fabricated graphitic carbon electrodes to act as electrochemical sensors has been investigated recently by our group and others [32,33]. Specifically, it was found that electrodes fabricated by 450 nm laser diode irradiation exhibited fast heterogeneous electron transfer rates (HET) ascribed to the highly porous and 3D morphology of the resulting structures, characterized by high surface area, large conductive networks and abundance of defect and edge plane, facilitating analyte adsorption and circulation [33]. As a result of their superior electrocatalytic properties, such electrodes enabled label-free concomitant detection of the biomarkers dopamine, ascorbic and uric acid, usually disabled in bulk electrodes by the overlapping of their oxidation potentials [33]. Moreover, various routes for the biomodification of graphitic carbon electrodes for biosensing applications have been developed recently. For example, Nayak et al. fabricated wearable biosensors and demonstrated detection of glucose in buffer, serum and blood [34]. Laser-scribed electrodes have been employed in food safety applications, showing an ability to detect biogenic amines in food samples [35], low levels of chloramphenicol antibiotic [36], and salmonella in chicken broth [37]. Importantly for diagnostic applications, the ability of graphitic carbon electrodes to detect thrombine via aptamer electrode modification, was recently demonstrated by Fenzl et al. [38]. The electrodes allowed fast (<1 h), sensitive and selective responses in buffer and serum, comparable and/or superior to other electrochemical detection methods available in literature. A new class of laser scribed nanostructured gold modified electrochemical sensing was recently realized by Rauf et al. The system was used as electrochemical aptasensor for detection of human epidermal growth factor receptor 2 (Her-2) and displayed ~2-fold enhancement in sensitivity and electrocatalytic activity compared to bare laser scribed electrodes and commercially available screen-printed gold electrodes [39].

In this paper, we present the use of laser scribed graphitic carbon electrodes for the detection of the inflammatory cytokine, IL-6. Electrodes were fabricated by a low-cost laser cutter tool equipped with a 450 nm wavelength laser, which led to formation of conductive porous structures with a high density of edge planes displaying spectral signatures of nanocrystalline graphitic carbon. The 3 W 450 nm laser cutting system offered significant cost reductions vs. high-powered CO_2_ systems, while retaining the high quality of graphitic material produced. The surface of the obtained electrodes was modified with anti-IL-6 recombinant antibodies selective for the inflammatory IL-6 cytokine. The assay was performed by monitoring the decreased electrochemical response (differential pulse voltammetry, DPV) to the redox probe [Fe(CN)_6_]^3−/4−^, associated to the reduced access of the probe to the anti-IL-6 recombinant antibodies-modified electrode following successful IL-6 binding. The resulting immunosensor response was linearly calibrated with log of concentration values between 10–500 pg/mL IL-6 concentration range and a low LOD in phosphate-buffered saline (PBS). The shelf-life stability was good, as detection responses were recorded for devices stored at 4 °C for six weeks. The faster time of analysis and lower cost compared to traditional ELISA methods make these immunosensors attractive for future diagnostic and point-of care applications.

## 2. Materials and Methods

### 2.1. Materials

Polyimide films (80 μm thickness) were purchased from Radionics, Ireland. Chemical reagents of 1-pyrenebutyric acid (PBA), potassium chloride, potassium ferrocyanide (K_4_[Fe(CN)_6_]), N-hydroxysuccinimide (NHS), 1-ethyl-3-(3-dimethylaminopropyl) carbodiimide (EDC) and bovine serum albumin (BSA), were purchased from Sigma-Aldrich, Ireland. Dulbecco’s phosphate saline (PBS) pH 7.4 and fetal bovine serum (FBS) were purchased from Gibco, Biosciences Limited, Ireland. Purified Rat Anti-IL-6, Recombinant Human IL-6 were purchased from BD Biosciences, UK. The IL-6 ELISA was sourced from R&D Systems, Bio-Techne, UK. All solutions were of analytical grade and were prepared using deionized Milli-Q water (resistivity 18.2 MΩ-cm) and deoxygenated with N_2_ prior to electrochemical analysis.

### 2.2. Electrode Fabrication

Graphitic carbon electrodes were fabricated as previously reported by raster scanning of designed electrode structures on polyimide by a KKmoon Compact Automatic Desktop Laser Engraving Machine equipped with a 3 W laser and illumination wavelength of 450 nm. The laser was operated at 30% power and 40% depth adjustment. After fabrication, electrodes were rinsed with acetone, isopropanol and deionised water to remove any residues from the laser engraving process.

### 2.3. Characterisation

The morphology of graphitic carbon electrodes was characterised by a cold-cathode field-emission scanning electron microscope (JEOL SEM, JSM-7500F, Jeol, Welwyn Garden City, UK) operating at 5 kV acceleration voltage. Raman measurements were performed with a Renishaw inVia Raman system (Renishaw, New Mills, UK) equipped with a 514 nm helium–neon laser. The laser beam was focused onto the sample through a Leica 20× objective with 0.4 N.A. Acquisition time was 10 s and measured power was 3 mW.

### 2.4. Electrode Modification

The surface of graphitic carbon electrodes was modified by immersion of the electrode in 1-PBA solution (DMSO, 250 mM) for 60 min. After rinsing with PBS, the electrode was immersed in an aqueous solution of 75 mM EDC:50 mM NHS for 90 min, followed by immersion in PBS for 15 min. Next, the graphitic carbon electrode was exposed to 200 µL of a 2.5 µg/mL PBS solution of Purified Rat Anti-Human IL-6 for 90 min followed by extensive washing with PBS. The unreacted electrode surface was blocked with BSA to reduce non-specific binding and subsequently rinsed with PBS, prior to IL-6 binding.

### 2.5. Electrochemical Analysis

Cyclic voltammetry (CV) and differential pulse voltammetry (DPV) electrochemical measurements were performed with an Autolab PGSTAT 302N potentiostat (Metrohm, Cheshire, UK) and hand-held EmStatblue (PalmSens, Gloucestershire, UK) electrochemical using a Pt wire as counter electrode, Ag/AgCl as reference electrode and graphitic carbon as working electrode. The three electrodes were assembled in a Teflon cell with a circular area of 8 mm diameter exposed to the electrolyte. CV experiments were carried out in 1 M KCl supporting electrolyte, 5 mM [Fe(CN)_6_]^4−/3−^ redox probe. The scan rates used for CV measurements were 10, 25, 50, 100 and 200 mV/s, in a potential range from −0.1 to 0.6 V. N_2_ was purged in the electrolyte solutions for 30 min prior to measurements.

### 2.6. Electrochemical Interleukin-6 (IL-6) Detection

Recombinant human IL-6 in 200 µL aliquots across a concentration range of 10–500 pg/mL in PBS was dispensed onto the antibody-modified graphitic carbon electrode for 90 min. After rinsing with PBS, DPV measurements were performed using [Fe(CN)_6_]^3−/4−^ (0.01 V/s scan rate, 0.025 V modulation amplitude, 0.005 V step potential, 0.5 s interval time and 0.05 s sample width). Interference studies were performed in 10% BSA solution (PBS) in the presence of 5 mM [Fe(CN)_6_]^4−/3−^ redox probe. To mimic clinical testing conditions, samples were examined in 10% FBS (10% w:w in PBS). For both testing scenarios, the electrode was immersed in solutions containing IL-6 at concentrations from 0 (blank) to 500 pg/mL for 90 min. After rinsing with PBS, DPV measurements were performed as described.

## 3. Results and Discussion

**Scheme 1:**
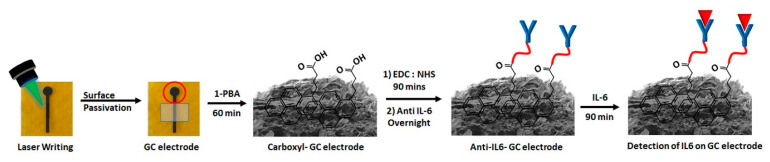
Schematic of graphitic carbon (GC) electrode fabrication, surface modification and interleukin-6 (IL-6) capture.

The fabrication and surface modification of graphitic carbon electrodes, and the detection mechanism of IL-6 cytokine, are shown in Scheme 1. Briefly, graphitic carbon electrodes of a “lollipop” shape were fabricated by laser irradiation of commercial polyimide tapes, as described previously [33]. Prior to chemical modification, the “lollipop” leg was passivated with nail varnish to ensure selective modification of the surface. The circular part of the electrode was then functionalised with 1-PBA, followed by anti-IL-6 attachment via EDC:NHS coupling. Finally, the electrode was incubated in the targeted IL-6 cytokine solution for 90 min prior to electrochemical detection.

**Figure 1:**
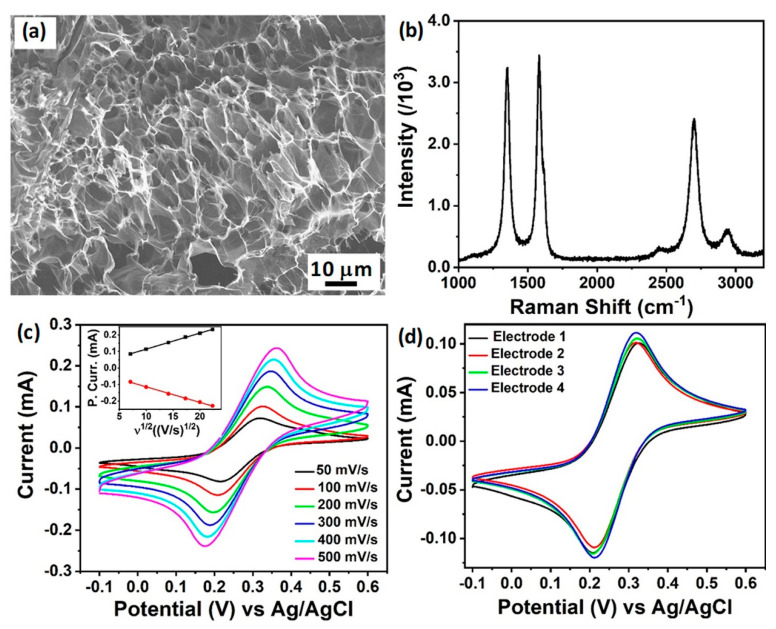
(**a**) Scanning electron microscope (SEM) image of a typical graphitic carbon electrode; (**b**) Raman spectrum of graphitic carbon electrodes; (**c**) cyclic voltammograms of graphitic carbon electrodes of 5 mM [Fe(CN)_6_]^3−/4−^ in 1 M KCl; Inset: peak oxidation and reduction current values vs. square root of potential scan rate; (**d**) cyclic voltammograms of graphitic carbon electrodes of 5 mM [Fe(CN)_6_]^3−/4−^ in 1 M KCl for four different electrodes.

Figure 1a shows a SEM image of the graphitic carbon surface, which was characterized as a flaky structure displaying high porosity and high surface area. Importantly for electrochemical analysis, the electrode surface showed the formation of an extended 3D network of graphitic carbon sheets with a high density of accessible edges constituted by kinked and wrinkled areas. The Raman spectrum of the graphitic carbon electrode (Figure 1b) was characterized by three main peaks centered at 1349, 1581 and 2696 cm^−1^ corresponding to the D peak (associated to bent sp^2^ carbon bonds and with the presence of defects), G peak (E2g vibration mode of graphitic carbon) and 2D peak, respectively. More specifically, the maximum in the *D* region could be fit with a single, sharp Lorentzian with full-width at half-maximum intensity, *FWHM*(*D*)~47 cm^−1^, consistent with low disorder. The ratio I_D_/I_G_ ≈ 1 confirmed the crystalline nature of the graphitized surface and was consistent with the formation of nanocrystalline graphitic domains in a disordered carbon matrix [40]; the high I_2D_/I_G_ ratio (0.78) indicated a low number of graphene layers [31,40]. Moreover, the 2D peak could be fitted by a single Lorentzian peak centered at 2696 cm^−1^, with *FWHM*(*2D*) of 81 cm^−1^. This profile was consistent with 2D graphene-like carbon structures consisting of randomly stacked graphene layers along the c-axis [31]. The electrochemical response of the graphitic carbon electrodes to inner sphere redox mediator [Fe(CN)_6_]^3−/4−^ was investigated in detail, key for future biosensor performance. Figure 1c shows the cyclic voltammograms recorded in 5 mM [Fe(CN)_6_]^3−/4−^ in a 1 M KCl supporting electrolyte in the interval 50–500 mV/s scan rates. The electrodes displayed a quasi-reversible behaviour, shown by the linear relationship between the peak oxidation/reduction current and the square root of the scan rate (inset Figure 1c) and indicated a semi-infinite linear diffusion reaction process with correlation coefficients for oxidation and reduction processes greater than 0.99. The average peak separation, ΔE_p_, calculated over four electrodes at 100 mV/s scan rate, was 112 mV (σ = 5.8 mV). The HET constant k^0^_app_ was calculated as 1.3 × 10^−1^ cm/s (σ = 1.8 × 10^−2^ cm/s; n = 4), as determined by the Nicholson method (see Figure S1 for details) [41]. This k^0^_app_ value was over one order of magnitude higher than values reported for other graphitic carbon electrodes obtained by direct laser writing [32,33,37]. In order to determine the contribution of the high porosity/surface area of the laser scribed material, a comparison between the electrode geometric area and the electrochemically active area (calculated using the Randles–Sevcik equation) was carried out [42]. The electroactive surface area (ESA, 11 mm^2^) was approximately 22% higher than the estimated geometric area (9 mm^2^), indicating the significance of the porous nature of the electrode material. The electrochemical behaviour was also tested over four electrodes (Figure 1d), which showed high reproducibility of electrochemical performance, key for the development of future reliable and stable biosensors.

**Figure 2:**
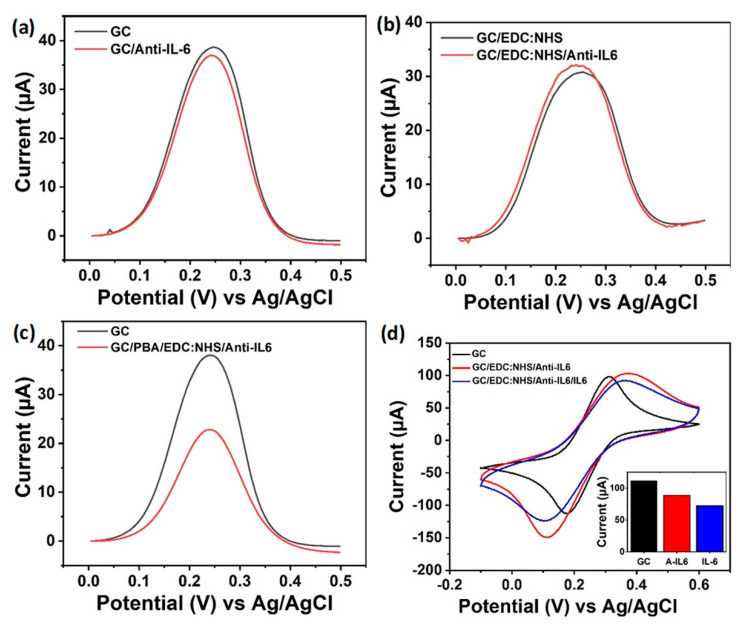
(**a**) Differential pulse voltammetry (DPV) curves of graphitic carbon (GC) electrode before and after attachment of anti-IL-6 antibody; (**b**) GC electrodes modified with EDC:NHS (1-ethyl-3-(3-dimethylaminopropyl) carbodiimide:N-hydroxysuccinimide) before and after anti-IL-6 attachment; (**c**) DPV curves of GC electrode with 1-PBA (1-pyrenebutyric acid) and EDC:NHS modification before and after attachment of anti-IL-6 capture antibody via EDC:NHS coupling; (**d**) cyclic voltammograms showing all different stages of GC electrode modification; all electrochemical measurements were performed in phosphate-buffered saline (PBS) at pH 7.4 containing 5 mM [Fe(CN)_6_]^3−/4−^.

Prior to the investigation of biosensing performance, preliminary studies were carried out in order to assess the capabilities of the unmodified graphitic carbon surface to work effectively as a biosensor. The DPV signal in response to [Fe(CN)_6_]^3−/4−^ in 0.1 M PBS supporting electrolyte was used to gauge the effect of surface modification. Figure 2a compares the DPV signals recorded at a bare graphitic carbon (GC) electrode and an electrode deposited with anti-IL-6. No significant change in peak current was observed. Figure 2b compares the DPV responses at two GC surfaces functionalized with EDC:NHS, one of them with anti-IL-6 deposited on it. A slight increase in maximum current was observed at the electrode with anti-IL-6 deposited, contrary to the expected decrease. These data suggest that the electrodes are not suitable for direct anti-IL-6 modification, and that the concentration of −COOH groups at the electrode surface was too low to support anti-IL-6 modification via EDC:NHS coupling. This result is consistent with XPS data previously reported for this material which showed an atomic percentage of −COOH equal to 1.96% [33]. In order to introduce more −COOH surface groups, electrodes were treated with 1-PBA. After 1-PBA treatment, EDC:NHS modification was re-attempted. Figure 2c shows the DPV results for a bare electrode and a PBA/EDC:NHS-modified electrode with anti-IL-6 deposited. In this instance the anti-IL-6 deposition led to a large drop in peak current (14.61 µA). This indicates the successful EDC:NHS-mediated attachment of anti-IL-6 to the electrode surface—the lower DPV response being attributed to lower accessibility of analyte to the modified electrode surface.

The optimal concentration of 1-PBA (250 mM) was determined by monitoring the access of the redox marker [Fe(CN)_6_]^3−/4−^ to the electrode surface for 1-PBA concentrations ranging from 10 to 500 mM in comparison to the response to the bare electrode. 1-PBA treatment of the electrode surface should introduce negatively charged −COOH groups by π–π interaction, resulting in electrostatic repulsion of [Fe(CN)_6_]^3−/4−^ [43]. This would cause a reduction of peak currents, and a retardation of the reaction (larger peak separations). Cyclic voltammograms (see Figure S2) showed that the initial peak current associated to the bare graphitic carbon electrode was as high as 104 μA, due to the unhindered response of [Fe(CN)_6_]^3−/4−^. Progressive increases in 1-PBA concentrations on the graphitic carbon electrode resulted in decreased maximum current to 32.52 μA and an overall spreading of the peaks was additionally observed, consistent with the increase in −COOH surface coverage. The EDC:NHS ratio was optimized by varying the concentrations, as reported in Figure S3. Only after exposure to 75 mM EDC:50 mM NHS did the graphitic carbon electrode show a decrease of the peak current upon binding of IL-6, proving that successful binding of anti-IL-6 via EDC:NHS coupling had occurred. The concentration of anti-IL-6 was chosen by monitoring the change of current intensity of various electrodes upon binding of different anti-IL-6 concentrations. Figure S4 showed that the highest differential current in DPV was obtained with 2.5 μg/mL anti-IL-6 concentration, which was then the concentration employed throughout this work. Figure 2d shows CV curves for [Fe(CN)_6_]^3−/4−^ with optimized conditions. Progressive blocking of the electrode surface, first by the attachment of anti-IL-6 and then by IL-6 to the graphitic carbon electrodes, leads to a progressive dropping of the peak current, as seen in the histogram inset of Figure 2d displaying the trend of peak current progression (background subtracted—a much larger capacitive current is observed for the modified electrodes, obscuring the fact that the peak current has indeed dropped).

**Figure 3:**
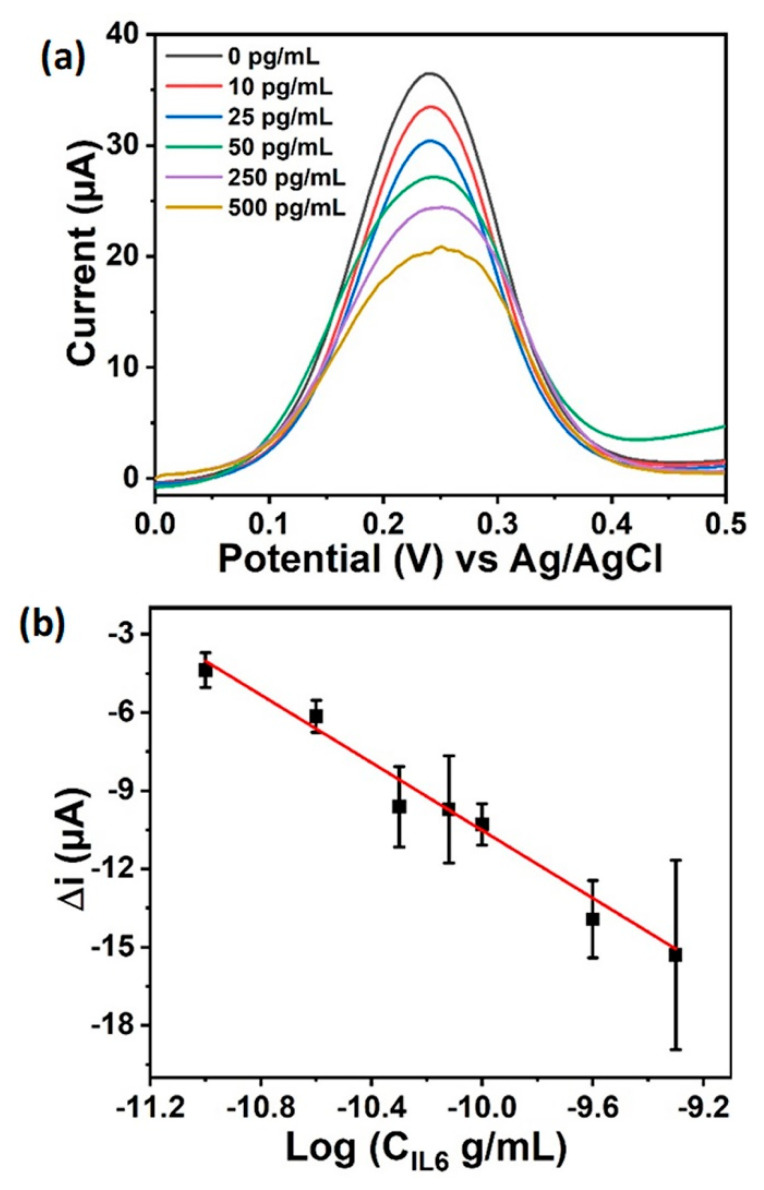
(**a**) DPV curves of graphitic carbon (GC) biosensor at varying concentrations of IL-6 conducted in PBS at pH 7.4 containing 5 mM Fe(CN)_6_]^3−/4−^; (**b**) Change of the DPV peak current of GC biosensor vs Ag/AgCl at varying IL-6 concentration, n = 3.

In order to test the capability of the fabricated biosensor for IL-6 detection, graphitic carbon working electrodes were biomodified as described above, assembled in a Teflon electrochemical cell and exposed to various IL-6 concentrations ranging from 10 to 500 pg/mL. Following an incubation time of 90 min, the electrochemical response of the electrode to [Fe(CN)_6_]^3−/4−^ was measured by DPV. Figure 3a shows the progressive decrease of DPV signal with the increase of IL-6 concentration, in agreement with the reduction of electrode surface available for the redox marker. Measurements were made in triplicate, and results showing the mean standard deviation are displayed in Figure 3b. The DPV peak currents decreased linearly with the log of the concentration according to the equation y = −75.4 − 6.5x, where y is current in μA and x is the log of IL-6 concentration in g/mL (R^2^ = 0.9738). From the data, it was calculated that the current drop was equal to −3.9 μA at 10 pg/mL IL-6 concentration and estimated as −1.95 μA at 5 pg/mL IL-6, a concentration value equivalent to the range found in healthy subjects. The LOD, calculated by the 3σ method, was calculated to be 5.1 pg/mL (see Supporting Information), therefore showing the potential of this immunosensor for detection of elevated IL-6 concentrations related to inflammatory events.

The performance of the developed electrochemical assay was benchmarked against ELISA, the traditional method commonly used to assess the concentration of IL-6. Results of the ELISA test are reported in Figure S5 and displayed comparable LOD to the immunosensor. However, the time required for the performing of the ELISA test as per the manufacturer’s instructions was 2 h, followed by a further 2 h of labelling/substrate incubation, substantially longer than the sub-90 min demonstrated by the present method.

The performance of the developed sensor was also compared with other electrochemical sensors reported in literature as shown in Table 1. The developed graphitic carbon sensor displayed a comparable LOD to most IL-6 sensors and at the same time offered the advantage of low-cost materials and fabrication methods. In fact, the sensor demonstrated low cost (estimated cost ca. €2 per test based on cost of chemicals used) and the technology presented offers easy routes for upscaling of production and development of on-chip and personalised healthcare solutions.

**Figure 4:**
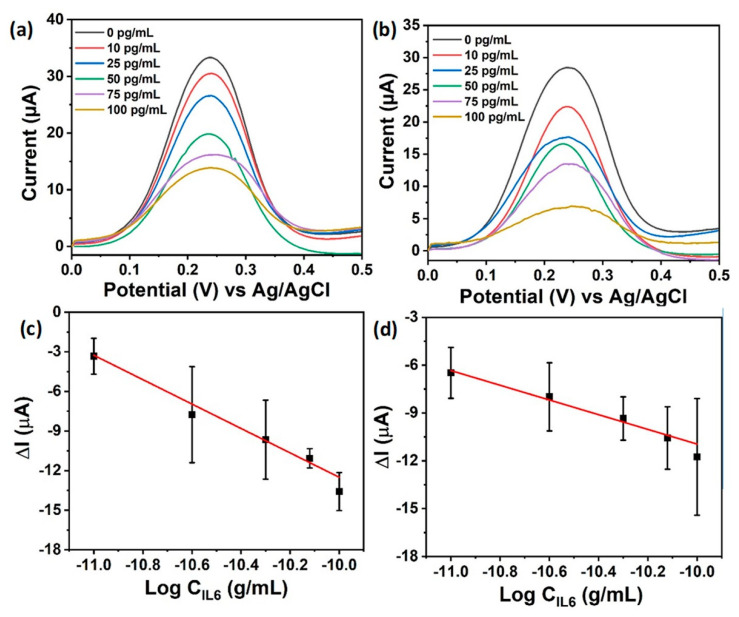
DPV curves of graphitic carbon biosensor at varying concentrations of IL-6 conducted in 0.1 M PBS at pH 7.4 containing 5 mM Fe(CN)_6_]^3−/4−^ in (**a**) 10% serum and (**b**) 10% BSA; change of the DPV peak current of GC biosensor vs. Ag/AgCl at varying IL-6 concentration in (**c**) 10% serum and (**d**) 10% BSA, n = 3.

In order to further investigate the performance of our biosensor platform towards real world analysis, DPV measurements were performed in PBS containing 10% serum (FBS) and 10% interferent BSA, respectively. The FBS study was carried out because cells grow in vitro in protein media with 10% serum. Besides, FBS contains other nutrients and supplement facilitating cell growth which can potentially affect the binding of IL-6. The BSA interference study was performed as albumin makes up 60% of the total protein found in blood. Figure 4a shows a linear decrease in differential current with the log of IL-6 concentration (Log[C]) in the range 10–500 pg/mL. A linear decrease of DPV peak currents with Log[C] was observed, described by the equation y = −105.0 − 9.2x was obtained (y is current in μA; x is log[C], C in g/mL; R^2^ = 0.9738, Figure 4c). Figure 4b shows the DPV response of the graphitic carbon electrode exposed to 10% BSA interferent concentration. Upon addition of IL-6, decrease of the DPV signal was observed, proving that the sensor maintained its selectivity to IL-6. The linear decrease of DPV peak currents with Log[C] followed the equation y = −57.10 − 4.6x (R^2^ = 0.9745, Figure 4d). In the presence of interferents the modified electrode maintains a characteristic current-Log[C] response. This data demonstrate the high robustness of the IL-6 antibody-modified graphitic carbon as a biosensor platform. The calibration of the response is variable and medium-sensitive. This variability is best captured by the changing sensitivity parameter (slope of calibration curve): −6.5 in PBS; −9.2 in 10% serum; −4.6 in 10% BSA. Interestingly, the sensitivity increased in the 10% serum matrix. The usable concentration range has decreased in both cases vs. PBS—the peak current at 500 pg/mL IL-6 in PBS is higher than at 100 pg/mL in serum and BSA (compare Figure 4a,b with Figure 3a).

**Figure 5:**
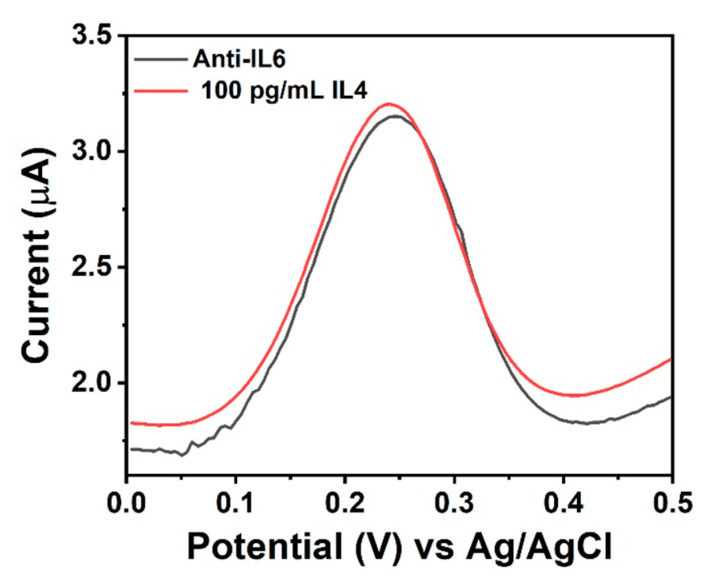
DPV curves of graphitic carbon biosensor (black curve) and biosensor exposed to 100 pg/mL of IL4 (red curve). DPV conducted in PBS at pH 7.4 containing 5 mM Fe(CN)_6_]^3−/4−^.

The selectivity of the immunosensor was examined by measuring its electrochemical response (DPV) to interleukin-4 (IL-4), produced mainly by mast cells and involved in immune regulation and wound repair [50]. As the surface of the electrode was modified with antibodies specific for IL-6, no current decrease was detected by DPV upon incubation of the biosensor in PBS containing IL-4 (Figure 5).

The stability of shelf life of the developed immunosensors was tested by measuring its current response by DPV after storage at 4 °C for 6 weeks. The mean change in current calculated for three sensors at IL-6 concentrations of 100 pg/mL was 9.61 μA, very close to the average time zero value of 10.3 μA (Figure S6).

**Figure 6:**
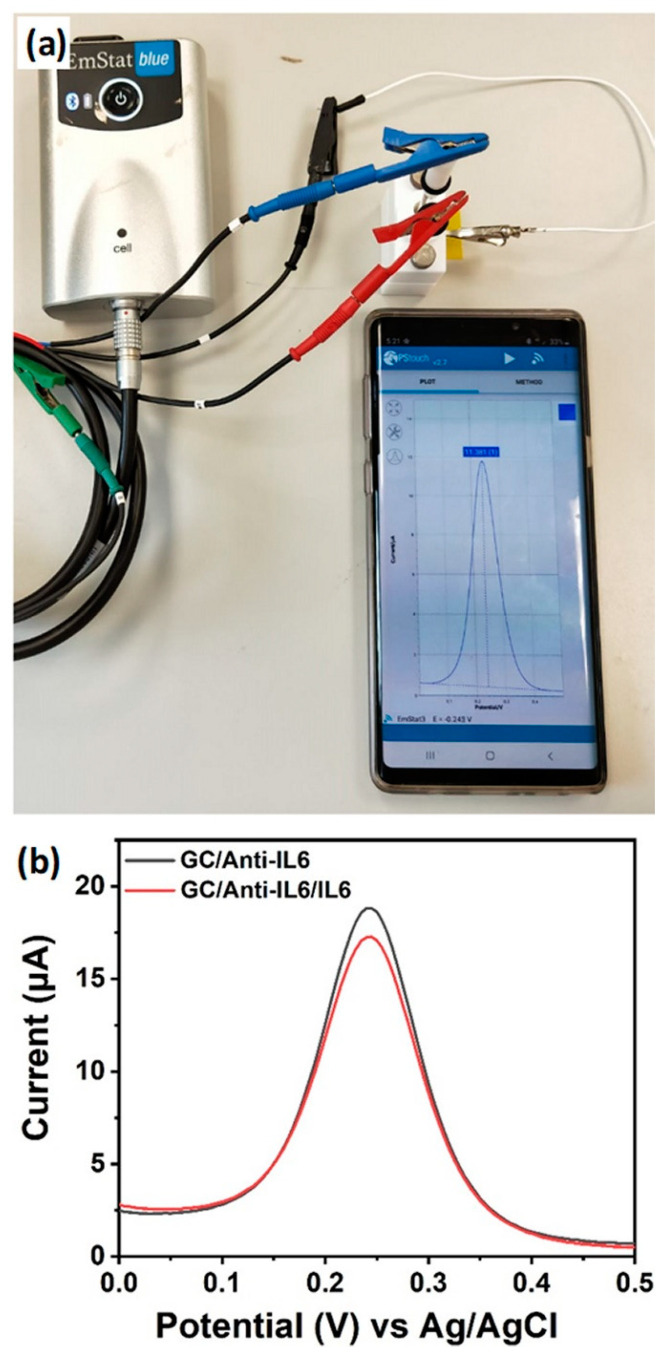
(**a**) Photograph of setup used for detection of IL-6 with handheld instrumentation; (**b**) DPV curves recorded with handheld instrumentation of 10 pg/mL IL-6 conducted in PBS at pH 7.4 containing 5 mM Fe(CN)_6_]^3−/4−^.

Finally, the immunosensor response was tested with hand-held instrumentation, in order to test the capability of miniaturisation for future point-of-care applications. Figure 6a shows the setup used with Bluetooth-operated mobile phone interface. Figure 6b shows the DPV curves for the graphitic carbon electrode with capture anti-IL-6 and the same electrode exposed to IL-6. Upon exposure to 10 pg/mL IL-6 a decrease in current, ΔI, equal to −1.6 μA was measured, showing the usability of hand-held instrumentation for point-of-care diagnostic analyses.

## 4. Conclusions

In conclusion, an electrochemical immunosensor for fast and selective detection of inflammatory IL-6 was developed based on the use of bio-modified graphitic carbon electrodes. The electrochemical performances were associated with the unique morphology of the electrode, displaying a highly porous, 3D and highly defective structure rich in edge planes. The immunosensor was able to detect IL-6 in the linear range 10–500 pg/mL with an LOD (n = 3) of 5.1 pg/mL in PBS. The modified electrodes maintained a linear current-Log[C] response in varying media. The high stability over time, selective response, relative fast analysis times, low cost and potential fabrication scalability make this immunosensor suitable for future lab-on-chip diagnostic applications. The compatibility with hand-held commercial instrumentation suggests suitability for point-of-care applications.

## Figures and Tables

**Table 1:** Comparison of different electrochemical biosensors for IL-6 detection *.

Material	Transducer	Detection Technique	Measured Range (pg/mL)	Detection Limit (pg/mL)	Medium	[Ref.]
SWNT/GSH-AuNPs	PG	CA	20–4000	10	Serum	[44]
FC-PPN	GC	SWV	2–20,000	1	PBS	[45]
Au NP	Gold	SWV	5–50,000	2	PBS	[19]
Ppy/Au NPs	SPGEs	EIS	1–15	0.3	PBS	[18]
SWCNTs	PG	CA	50–500	30	Human serum	[46]
Au NP ink	Inkjet electrodes	CA	20–40	20	Calf serum	[17]
(Au NP)-graphene-SiO_2_	ITO	CA	1–40	0.3	PBS	[16]
Au NPs/SWCNTs	SiO_2_/Si	EIS	10–10^7^	10	Serum	[6]
Ag NPs	ERGO-AuPdNPs	EIS	10^2^–10^9^	59	PBS	[47]
SWNTs	BPPG	CA	–	0.5	Calf serum	[48]
SWCNTs	FET	I-V	1–100	1.37	PBS	[49]
Graphitic carbon	Multilayer graphene	DPV	10–500	5	PBS	This work

* SWNT = single wall carbon nanotubes; GSH-AuNP = Glutathione-protected gold nanoparticles; Ppy = polypyrrole; FC-PPN = ferrocene loaded porous polyelectrolyte; PG = pyrolytic graphite; SPGEs = screen printed graphite electrodes; ERGO-AuPdNPs = reduced graphene oxide and gold−palladium bimetallic nanoparticles; BPPG Basal plane pyrolytic graphene; FET field effect transistor; CA = chronoamperometry; SWV = square wave voltammetry; LSV = linear sweep voltammetry.

## Data Availability

\FS1\Docs4\daniela.iacopino\My Documents\Projects\Laserflame\.

## Supplementary Materials

The following are available online at https://www.mdpi.com/article/10.3390/nano11082110/s1, Figure S1: HET transfer coefficient (k^0^) calculation, Figure S2: Optimization of 1-PBA concentration, Figure S3: Optimization of NHS:EDC coupling conditions, Figure S4: Optimization of anti-IL6 concentration conditions. Limit of detection calculation, Figure S5: Benchmarking against ELISA test, Figure S6: Biosensor shelf life.
