# Supplementary material for: Laser Scribing Fabrication of Graphitic Carbon Biosensors for Label-Free Detection of Interleukin-6
Source: Nanomaterials (Basel). 2021 Aug 19;11(8):2110. doi: 10.3390/nano11082110 (PMC8399033; doi:10.3390/nano11082110)
Supplement: Supplementary file 1 [file nanomaterials-11-02110-s001.zip › nanomaterials-1321516-supplementary.pdf]

Supporting information

Laser Scribing Fabrication of Graphitic Carbon Biosensors for Label-Free Detection of Interleukin-6

Pei Shee Tan [1,2,†], Eoghan Vaughan [3,†], Jahidul Islam [3], Niall Burke [1], Daniela Iacopino [3,*] and Joanna B. Tierney [1,2]

[1] Shannon Applied Biotechnology Centre, Munster Technological University, Tralee, V92KA43, Kerry, Ireland; Pei.Shee.Tan@research.ittralee.ie (P.S.T.); Niall.Burke@staff.ittralee.ie (N.B.); Joanna.Tierney@staff.ittralee.ie (J.B.T.)
[2] Department of Biological and Pharmaceutical Sciences, Munster Technological University, Tralee, V92KA43, Kerry, Ireland
[3] Tyndall National Institute, University College Cork, Dyke Parade, T12R5CP, Cork, Ireland; eoghan.vaughan@tyndall.ie (E.V.); jahidul.islam@tyndall.ie (J.I.)
* Correspondence: daniela.iacopino@tyndall.ie
† These authors contributed equally.

Heterogeneous Electron Transfer (HET) coefficient rate calculation

The HET rate (k^0) for fabricated graphitic carbon electrodes was calculated using the Nicholson method for $[Fe(CN)_6]^{3-/4-}$ redox mediator systems. The following equation was used to relate k^0 to the dimensionless kinetic parameter Ψ:[1]

$$\Psi = k^o \left(\frac{D_O}{D_R}\right)^{\frac{\alpha}{2}} \sqrt{\frac{RT}{n\pi F D_O \nu}} \tag{1}$$

where D_O and D_R are the diffusion coefficients of the oxidised/reduced forms of the analyte respectively, α is the charge transfer coefficient, n is the number of electrons involved in the electron transfer reaction, ν is the scan rate (Vs^{-1}), F is the Faraday constant, R is the universal gas constant and T is the absolute temperature. Equation (1) can be written more simply as:

$$\Psi = k^o C\nu^{-1/2} \tag{2}$$

where C is a constant characteristic of the given analyte under investigation. The following equation was used to relate the peak separation (ΔE_P) to k^o:[2]

$$\Psi = \frac{(-0.6288 + 0.0021\,X)}{(1 - 0.017\,X)} \tag{3}$$

where X is equal to ΔE_P (mV) multiplied by the number of electrons involved in the reaction.

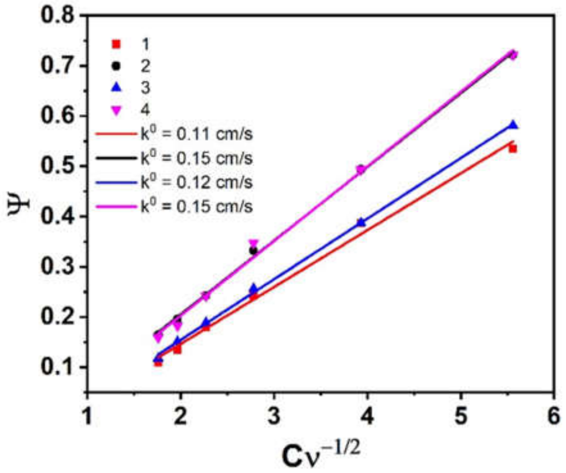

Figure S1. HET transfer coefficient (k^0) calculation for fabricated graphitic carbon electrodes using $[Fe(CN)_6]^{3-/4-}$ as redox mediator.

Optimization of 1-PBA concentration

Graphitic carbon electrodes were exposed to different concentration of 1-PBA, ranging from 10 to 500 mM. At low 1-PBA concentrations no decrease of the electrochemical response of $[Fe(CN)_6]^{3-/4-}$ was obtained, signifying that not enough –COOH groups were present on the electrode surface. The optimum concentration was selected from the decrease in intensity of electrochemical response registered at higher 1-PBA concentrations.

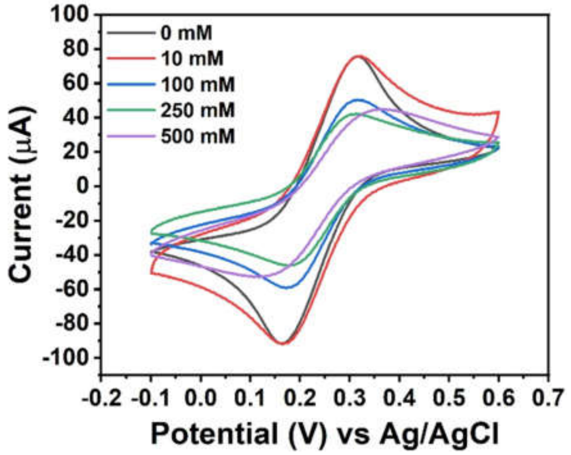

Figure S2. Cyclic voltammograms of 1 mM $[Fe(CN)_6]^{3-/4-}$ in 1 M KCl for graphitic carbon electrodes exposed to different concentrations of 1-PBA.

Optimization of NHS:EDC coupling conditions

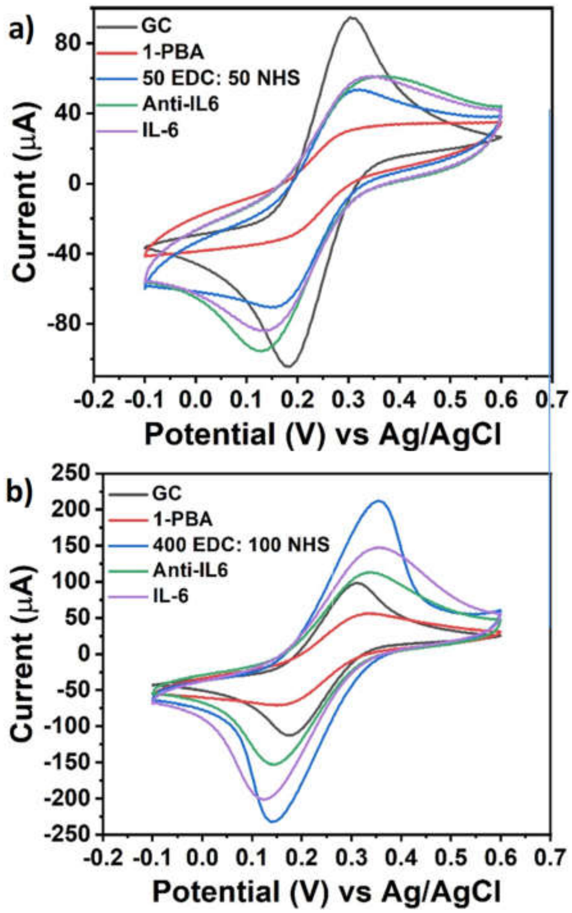

Figure S3. Cyclic voltammograms of 1 mM $[Fe(CN)_6]^{3-/4-}$ in 1 M KCl for graphitic carbon electrodes exposed to different ratios of EDC:NHS: a: 50 mM EDC: 50 mM NHS; b) 400 mM EDC: 100 mM NHS.

Optimization of anti-IL6 concentration conditions

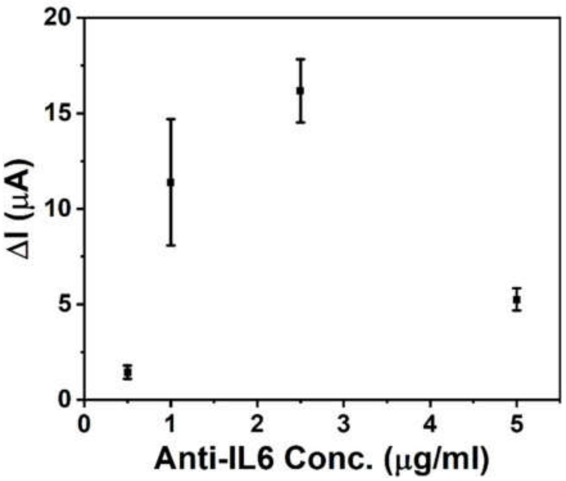

Figure S4. Differential current values obtained from cyclic voltammograms of 1 mM $[Fe(CN)_6]^{3-/4-}$ in 1 M KCl for graphitic carbon electrodes exposed to different concentrations of anti-IL6. Differential current values are the difference in current obtained for pure GC electrodes and GC electrodes exposed to anti-IL6.

Limit of detection calculation

The LOD was calculated using the 3σ method. This form of calculation is generally applied to a linear calibration curve, where the formula is simply

$$LOD = 3\sigma/m \quad (4)$$

where m is the slope of the calibration curve. The calibration curve presented in this paper is linear with respect to the Log of the concentration. The current values are differential and are negative. This means that the above formula must be modified: the y-intercept will be non-zero and must be considered, as the origin no longer represents a point of zero concentration and zero signal; *LOD* must be replaced by *Log(LOD)*, adding an extra calculation step; we must consider the negative value of σ:

$$Log(LOD) = (-3\sigma - c)/m \quad (5)$$

$$LOD = 10^{(-3\sigma - c)/m} \quad (6)$$

where c is the y-intercept of the calibration line. Equation (6) was used to calculate the value of the LOD for these sensors.

Benchmarking against ELISA test

The performance of fabricated electrochemical immunosensor was benchmarked against the traditional ELISA assay. The lowest IL6 concentration measurable by ELISA was 10 pg/mL. The testing time required labelling and 2 hours testing time, in contrast with label free and 90 min long immunosensor test response.

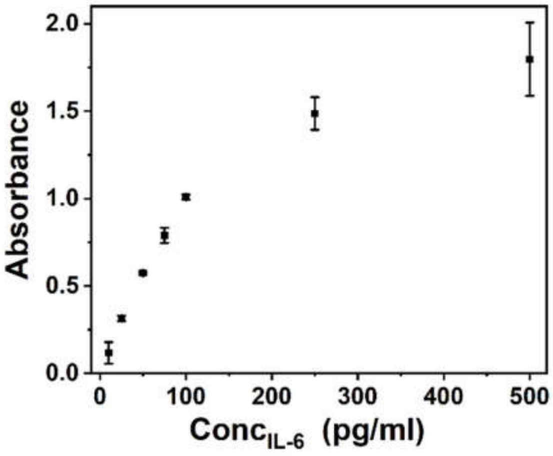

Figure S5. ELISA detection of IL6.

Biosensor shelf-life

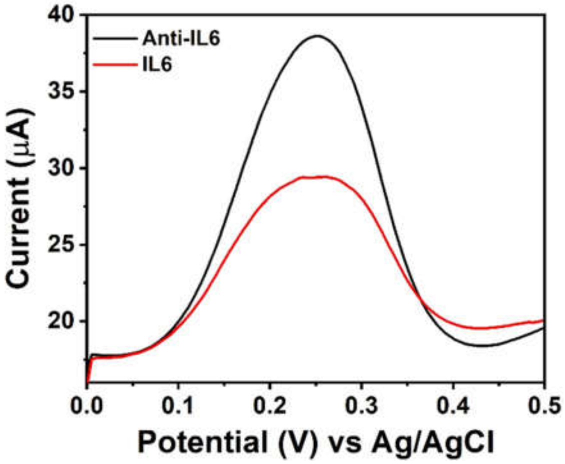

Figure S6. DPV curves of bare GC biosensor (black curve) and biosensor exposed to IL6 10 pg/mL following storage at 4 °C for six weeks (red curve). DPV conducted in PBS at pH 7.4 containing 5 mM $Fe(CN)_6]^{3-/4-}$.
